# Drug-Induced Lichenoid Photosensitivity: A Case Report

**DOI:** 10.7759/cureus.82777

**Published:** 2025-04-22

**Authors:** Shrinivas R Raikar, Sneha Sneha, Sreeraj G, Janarthanan Rajkumar

**Affiliations:** 1 Pharmacology, BLDE (Deemed to be University) Shri BM Patil Medical College Hospital and Research Centre, Vijayapura, IND

**Keywords:** adverse drug reaction, antitubercular drugs, multidrug resistance, rechallenge test, tuberculosis

## Abstract

Tuberculosis (TB) is a highly contagious airborne bacterial infection and continues to be one of the leading causes of mortality worldwide. First-line antitubercular therapy (ATT), including isoniazid, rifampicin, pyrazinamide, and ethambutol, is essential for TB management but is associated with adverse drug reactions (ADRs), including severe cutaneous manifestations. Managing these ADRs poses significant challenges, as discontinuation of ATT, combined with systemic steroid use, may increase the risk of disease progression and multidrug resistance. Drug-induced lichenoid photosensitivity is an uncommon yet clinically significant skin-related adverse reaction to ATT. Its unpredictable onset and clinical resemblance to autoimmune disorders make early recognition and accurate diagnosis essential to avoid mismanagement or delays in treatment. Timely identification and appropriate intervention are critical not only for minimizing complications but also for maintaining uninterrupted TB therapy. This case report highlights the importance of recognizing and effectively managing drug-induced lichenoid photosensitivity associated with antitubercular agents. We describe a 62-year-old male diagnosed with cervical tuberculous lymphadenitis who developed this rare cutaneous adverse reaction during ATT. The case underscores the need for early diagnosis, individualized treatment strategies, and the vital role of pharmacovigilance in promoting patient safety and ensuring the uninterrupted continuation of TB management.

## Introduction

Drug-induced lichenoid photosensitivity is a rare but clinically important cutaneous adverse reaction to antitubercular therapy (ATT). An estimated 10 million people suffer from it annually [1]. The National Guidelines for Tuberculosis Diagnosis and Management state that the use of particular antitubercular drugs that target Mycobacterium tuberculosis (TB) is the mainstay of TB treatment. The first-line ATT includes rifampicin, isoniazid, pyrazinamide, and ethambutol [2]. Cutaneous adverse drug reactions (ADRs) are well-known undesired effects of these drugs and vary from mild pruritus to life-threatening toxic epidermal necrolysis, which requires discontinuation of the treatment [1].

ADRs can be predictable or unpredictable, but in either case, they must be communicated to patients to enhance healthcare quality. According to WHO, TB was the 13th leading cause of death worldwide in 2020. Clinicians frequently encounter TB, and the effectiveness of antitubercular drugs such as rifampicin, isoniazid, pyrazinamide, and ethambutol can be compromised by the severity of adverse reactions. This necessitates careful consideration of both the benefits and risks of these medications, requiring physicians to select appropriate management strategies [3].

Lichenoid photosensitivity is an uncommon but recognized cutaneous adverse effect of antitubercular medications, especially rifampicin and isoniazid. It is a T-cell-mediated hypersensitivity reaction triggered by antigenic changes induced by UV exposure, leading to keratinocyte apoptosis and inflammation. A history of photosensitivity can be present, as ultraviolet radiation can induce a photochemical reaction. This reaction can lead the body to recognize the drug or its metabolites as non-self-antigens, triggering an immune response. The incidence of lichenoid photosensitivity emphasizes how critical it is to detect and eliminate the offending substance as soon as possible in order to avoid more problems. Furthermore, autoimmune disorders must be ruled out because they can manifest similarly to drug-induced responses [4]. Common drugs that can cause lichenoid reactions include captopril, enalapril, chloroquine, methyl dopa, and D-penicillamine. Isoniazid and rifampicin are two antitubercular medicines that are frequently linked to lichenoid drug eruptions. These drugs contribute to lichenoid eruptions by triggering delayed-type hypersensitivity reactions, leading to basal vacuolar degeneration, apoptosis of keratinocytes, and inflammation mediated by CD8+ T cells [3].

ADRs associated with TB often lead to interruptions and modifications in treatment, which can contribute to treatment failure, the development of drug resistance, relapse, and the ongoing transmission of the disease. The risk of transmission emphasizes the need to resume treatment as soon as possible; yet, the management procedure is made more difficult by the scarcity of effective anti-TB medications. TB is linked to a wide spectrum of severe cutaneous ADRs, including lichenoid drug reactions (LDRs), drug hypersensitivity syndrome, and Stevens-Johnson syndrome. In all cases, except for LDRs, acute clinical or laboratory markers facilitate early detection and prompt discontinuation of the offending drug following drug rechallenge [5].

This report aims to highlight the case of a 62-year-old male with a known case of cervical TB lymphadenitis who developed drug-induced lichenoid photosensitivity during treatment with antitubercular drugs at Shri BM Patil Medical College Hospital and Research Centre, Vijayapura, India.

## Case presentation

Patient information

A 62-year-old male farmer, with a known history of cervical TB lymphadenitis, had been on ATT with Tab. AKURIT-4 (rifampicin 150 mg, isoniazid 75 mg, ethambutol 275 mg, and pyrazinamide 400 mg) for the past three months. He had no history of smoking, alcohol consumption, or other comorbidities.

Clinical presentation

The patient developed pruritic rashes and hyperkeratotic lesions, which initially appeared on the arms and legs but progressively spread across the body. On general examination, he was moderately built and nourished, conscious, cooperative, and well oriented to time, place, and person.

**Figure 1:**
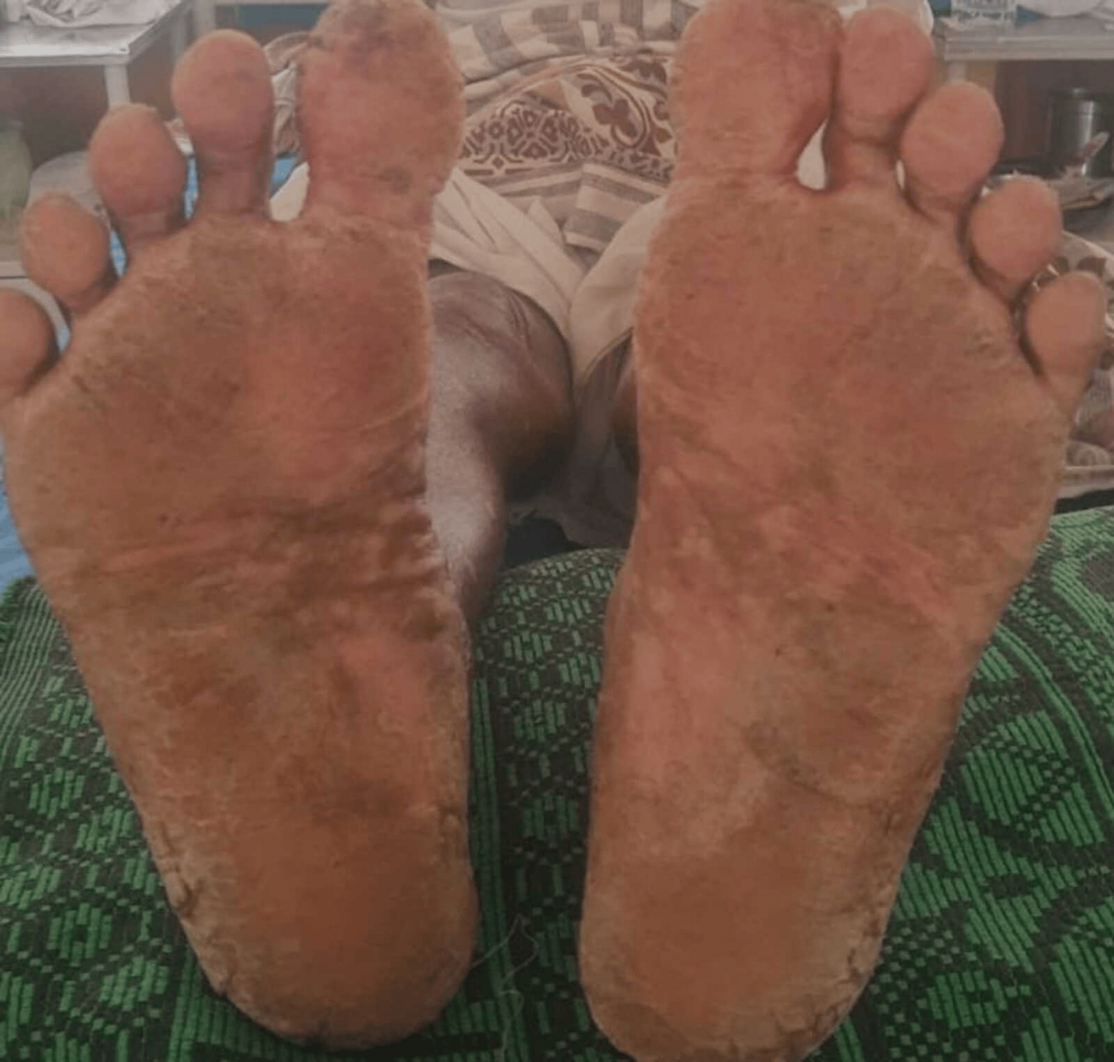
Hyperkeratotic plaques with fissures present over the plantar aspect of both feet

A solitary, firm, non-tender left cervical lymph node was palpable. Pitting pedal edema was observed over the right lower limb, extending up to the knee, which was not associated with pain. There were no signs of pallor, icterus, cyanosis, clubbing, or facial edema. The patient denied any history of joint pain, burning micturition, or abdominal pain. Hyperkeratotic plaques with fissures present over the plantar aspect of both feet are shown in Figure 1.

Vital signs and systemic examination

The patient’s vital signs were within normal limits, with a blood pressure of 120/80 mmHg, a pulse rate of 78 bpm, a temperature of 98.6°F (afebrile), and a weight of 55 kg. Systemic examination of the cardiovascular, central nervous, respiratory, and abdominal systems did not reveal any abnormalities.

Dermatological examination

**Figure 2:**
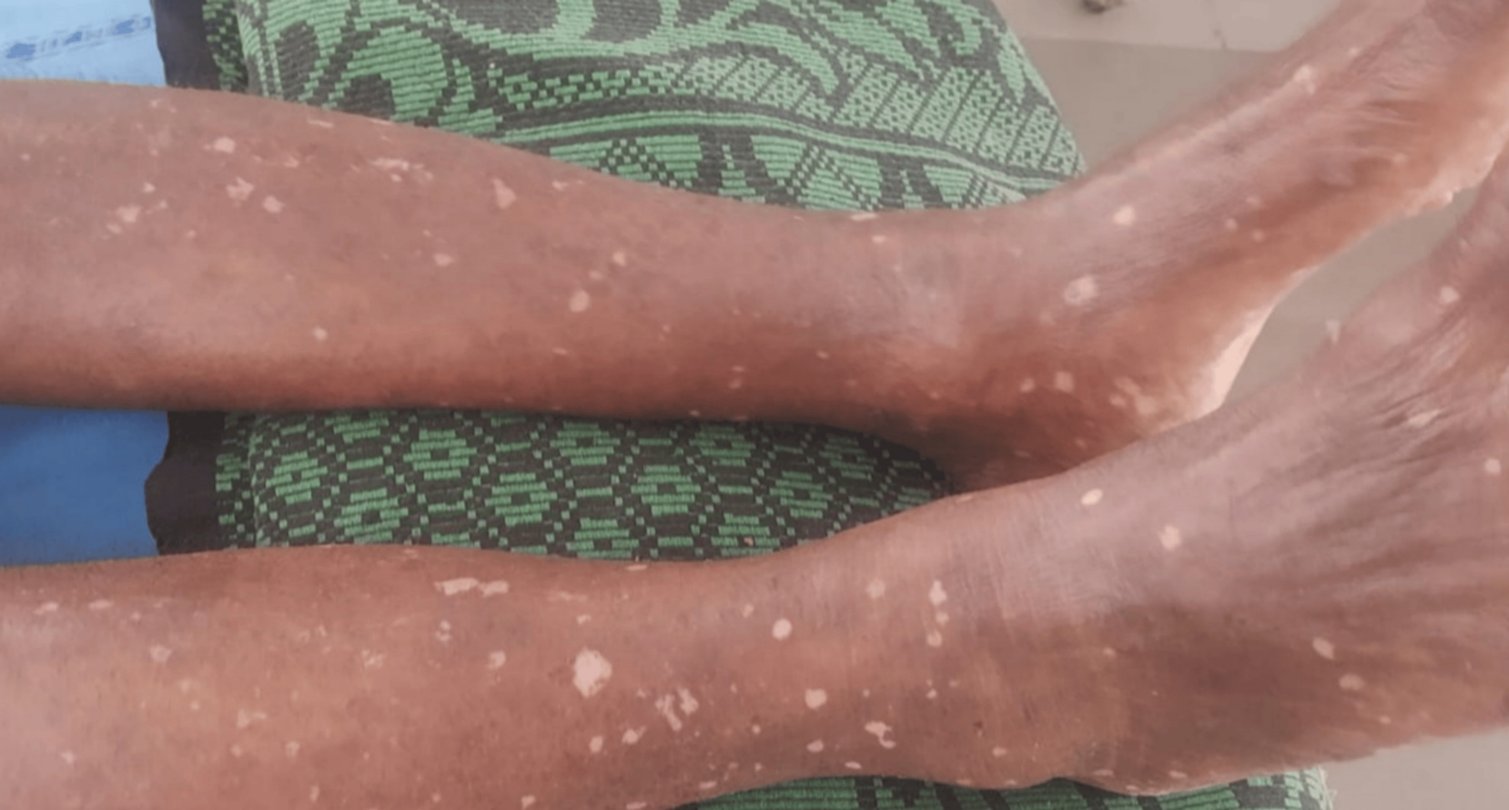
Multiple erythematous, hypopigmented macules over bilateral lower limb extremities

Multiple erythematous, hypopigmented macules, papules, and plaques were observed on the abdomen, bilateral upper limbs, and lower extremities. Multiple excoriations were present on the right lower extremity, bilateral upper limbs, and back. Hyperkeratotic plaques with superficial and deep fissures were noted on the plantar aspect of both feet. The mucosa and hair appeared normal. Nail examination revealed subungual hyperkeratosis in a few toenails, koilonychia of the right second and fourth toenails, and pitting of the left index fingernail. Multiple erythematous, hypopigmented macules over bilateral lower limb extremities observed in this patient are depicted in Figure 2.

Diagnostic assessment

Fine-needle aspiration cytology of the cervical lymph node, performed on November 5, 2022, revealed suppurative lymphadenitis. Ziehl-Neelsen staining for acid-fast bacilli was negative.

Laboratory investigations demonstrated relative neutrophilia of 84.8% (reference range: 40-70%) with lymphopenia of 7.0% (reference range: 20-40%), a hemoglobin level of 12.1 g/dL (reference range: 13.5-17.5 g/dL), and normocytic normochromic anemia. The platelet count was 252 × 10³/µL (reference range: 150-400 × 10³/µL), and platelet indices were within normal limits. The erythrocyte sedimentation rate was elevated at 39 mm/hr (reference range: 0-20 mm/hr), indicating an ongoing inflammatory process. Electrolytes and renal function tests were within normal limits.

The dermatological presentation prompted consideration of several differential diagnoses, including cutaneous lupus erythematosus, dermatomyositis, photodermatitis, and drug-induced lichenoid eruption. Autoimmune etiologies were considered less likely due to the absence of systemic manifestations, negative serological workup, and lack of mucosal involvement. The temporal relationship with ATT, the distribution of lesions on sun-exposed areas, and clinical improvement following drug withdrawal supported the diagnosis of drug-induced lichenoid photosensitivity. A skin biopsy and photopatch testing were not performed, as the clinical features and rechallenge response were sufficiently conclusive, and further delay in TB treatment was not warranted.

Treatment and management

Given the severity of the reaction, ATT was temporarily withheld, and systemic corticosteroid therapy was initiated to manage inflammation and prevent further progression. The patient received intravenous dexamethasone (8 mg daily) for five days, followed by oral prednisolone (40 mg daily) for two weeks. The prednisolone dosage was then tapered to 30 mg daily for an additional week.

Topical treatment was also implemented, including fluticasone propionate 0.05% cream and a 10% urea-based emollient. This combination resulted in significant improvement of the dermatological symptoms, including reduced erythema, scaling, and pruritus. The emollient also helped restore the skin barrier, improving hydration and alleviating dryness.

Following the completion of this regimen, the patient showed substantial improvement in both dermatological and overall clinical conditions. This allowed for the safe resumption of ATT, with ongoing monitoring to ensure therapeutic efficacy and prevent further complications.

Drug rechallenge and ADR assessment

To identify the offending drug, AKURIT-3 (excluding pyrazinamide) was reintroduced; however, the patient developed similar dermatological symptoms, suggesting an alternative causative agent. A stepwise drug rechallenge was subsequently performed. Isoniazid administration resulted in itching and facial edema, necessitating its discontinuation. Rifampicin was well-tolerated without any adverse reactions. Rifampicin was retained due to its essential role in TB treatment and its lower likelihood of causing lichenoid eruptions compared to isoniazid and ethambutol. The stepwise drug withdrawal approach allowed the identification of the causative agents. Ethambutol induced itching, fever, and facial edema within six hours of administration, leading to its discontinuation. After the discontinuation, the patient was transitioned to a second-line anti-TB regimen including levofloxacin to prevent treatment interruption.

To establish causality, the Naranjo Adverse Drug Reaction Probability Scale [6] was applied, as it is a standardized tool for evaluating ADRs. In this case, the Naranjo score was 7, classifying the reaction as “probable.” This assessment was based on key criteria, including the temporal association with drug administration, symptom resolution upon drug discontinuation, and recurrence following rechallenge. The individual scoring components were as follows: (1) prior conclusive reports of a similar reaction (+1); (2) adverse event occurring after drug administration (+2); (3) symptom improvement upon drug withdrawal (+1); (4) recurrence of symptoms upon rechallenge (+2); and (5) absence of an alternative explanation for the reaction (+1).

In this case, the diagnosis of drug-induced lichenoid photosensitivity was established based on clinical presentation, lesion morphology, and the temporal association with ATT. Although a biopsy was not performed, the characteristic cutaneous manifestations, resolution of symptoms upon drug withdrawal, and recurrence upon rechallenge provided strong clinical evidence supporting the diagnosis. While photopatch testing and UV provocation tests could have further corroborated photosensitivity, the clear exacerbation of lesions with sun exposure and subsequent improvement with photoprotection were considered sufficient for diagnostic confirmation. Given the need to ensure uninterrupted TB treatment and the absence of indications necessitating invasive procedures, additional investigations were not pursued.

Outcome and follow-up

The patient was continued on rifampicin along with second-line anti-TB drugs. The stepwise drug withdrawal approach allowed the identification of causative agents. Ethambutol and isoniazid were discontinued, and the patient was transitioned to a second-line anti-TB regimen including levofloxacin to prevent treatment interruption.

Upon completing the treatment regimen, the patient exhibited significant improvement in both dermatological and overall clinical conditions, facilitating the safe resumption of ATT. Continuous monitoring was implemented to ensure the effectiveness of the therapy and to mitigate any potential complications. Subsequent follow-up visits demonstrated further clinical progress, with complete resolution of the dermatological symptoms and no recurrence of adverse reactions. A comprehensive summary of the clinical events and management is provided in Table 1.

**Table 1:** Summary of clinical events and management ADR, adverse drug reaction; AKURIT-4, fixed-dose combination of isoniazid, rifampicin, ethambutol, and pyrazinamide used in the intensive phase of tuberculosis treatment; ATT, antitubercular therapy; ESR, erythrocyte sedimentation rate; FNAC, fine-needle aspiration cytology; TB, tuberculosis; ZN, Ziehl-Neelsen

Stage	Details
Patient information	62-year-old male with cervical tuberculous lymphadenitis, on antitubercular therapy (AKURIT-4) for three months
Symptoms	Presented with pruritic rashes, hyperkeratotic plaques, erythematous macules, excoriations, cervical lymphadenopathy, and pitting pedal edema
Investigations	FNAC showed suppurative lymphadenitis; ZN staining was negative. Laboratory tests revealed neutrophilia (84.8%; reference range: 40-70%), lymphopenia (7.0%; reference range: 20-40%), a hemoglobin level of 12.1 g/dL (reference range: 13.5-17.5 g/dL), and an ESR of 39 mm/hr (reference range: 0-20 mm/hr).
Management	ATT was discontinued due to suspected adverse drug reaction. Treatment included IV dexamethasone, oral prednisolone, topical fluticasone, and emollients.
Drug rechallenge and ADR assessment	Isoniazid and ethambutol reintroduction triggered adverse reactions and were discontinued. Rifampicin was well tolerated and continued alongside second-line anti-TB medications. Naranjo ADR score was 7, indicating a probable adverse drug reaction.
Outcome and follow-up	The patient showed progressive clinical improvement with no further adverse events reported during follow-up.

## Discussion

TB treatment is associated with a broad range of severe cutaneous ADRs, including LDRs, drug hypersensitivity syndrome, and Stevens-Johnson syndrome. Identifying and managing these complications is critical to maintaining treatment adherence. ATT is known to cause a spectrum of cutaneous ADRs. However, the absence of acute biochemical markers and the gradual onset of lichenoid drug eruptions complicate the establishment of a temporal relationship and causality assessment. The underlying mechanism involves T-cell expansion in response to the drug, triggering a delayed-type IV hypersensitivity reaction. Several factors contribute to susceptibility, including genetic predisposition, advanced age, female sex, diabetes, organ dysfunction, polypharmacy, infections (e.g., HIV and EBV), autoimmune conditions (e.g., rheumatoid arthritis, Sjogren’s syndrome, and systemic lupus erythematosus), hematological malignancies, and fixed-dose combinations of antitubercular drugs. Key mediators implicated in this reaction include INF-α, IFN-γ, and CXCR3 ligands. The primary treatment involves high-potency topical corticosteroids, with the latency period for symptom onset ranging from 15 days to six months [7].

The delayed onset of symptoms following prolonged ATT use presents a significant diagnostic challenge. Furthermore, the clinical features often mimic autoimmune disorders such as lupus erythematosus and dermatomyositis, necessitating careful differential diagnosis. Drug-induced lichenoid photosensitivity is exceedingly rare, with only sporadic cases reported in the literature from India. This rarity underscores the clinical significance of the present case, highlighting the need for heightened awareness and prompt recognition of such ADRs to ensure appropriate management.

LDRs, mediated by type IV hypersensitivity and TNF-α activation, lead to keratinocyte apoptosis via CD8+ T cells, resulting in inflammation, hyperpigmentation, and characteristic histologic changes such as saw-tooth rete ridges. While a biopsy is often required in atypical cases, the diagnosis in this case was established based on clinical history, lesion morphology, and recurrence upon rechallenge [8]. Photosensitivity was diagnosed clinically, as photopatch testing and UV provocation tests were not performed. The clear temporal association between sun exposure and lesion exacerbation, along with symptom improvement following photoprotection, provided sufficient evidence to support the diagnosis.

A review of the literature reveals several cases that mirror our findings, reinforcing the clinical relevance of ATT-induced lichenoid reactions. Singh et al. (2020) reported a 63-year-old male on ATT who developed violaceous, pruritic plaques on the extremities after four months of therapy. A structured drug rechallenge identified isoniazid as the causative agent, with symptom recurrence upon reintroduction and improvement following corticosteroid therapy [9].

Ali (2020) described a 55-year-old woman who developed pruritus and lichenoid lesions on sun-exposed areas after initiating fixed-dose ATT. The rechallenge confirmed isoniazid sensitivity; the drug was discontinued, and the patient improved with symptomatic treatment while continuing the remaining regimen [10].

Seo et al. (2016) presented a 73-year-old woman with generalized lichenoid papules and histologic features of lichen planus. Lesions worsened with ethambutol rechallenge and resolved after its withdrawal, confirming it as the offending agent [11].

A recent report by Shanmukhappa et al. (2024) identified ethambutol as the likely causative agent in a case of lichenoid drug eruption. Rechallenge with isoniazid and rifampicin caused no recurrence, and the patient’s lesions resolved gradually with supportive therapy. Although ethambutol is commonly associated with optic neuritis and peripheral neuropathy, its link to lichenoid eruptions is rarely reported. Treatment involved corticosteroids, antihistamines, and phosphodiesterase inhibitors [12].

These cases, along with ours, show consistent patterns such as delayed symptom onset, sun-exposed involvement, and the utility of drug rechallenge for identifying the offending agent - most often isoniazid or ethambutol. Given the clinical resemblance to autoimmune dermatoses like lupus and dermatomyositis, diagnosis relies on temporal association, lesion morphology, and treatment response. These findings highlight the need for clinical vigilance, individualized rechallenge protocols, and strong pharmacovigilance to support early diagnosis and uninterrupted TB care.

ADRs, especially cutaneous ones, require a structured approach to identify and manage. Tools like the Naranjo Probability Scale and WHO-Uppsala Monitoring Centre Causality Assessment System are commonly used to assess the likelihood of a drug causing an ADR. In this case, the Naranjo ADR Probability Scale [6] classified the reaction as “probable,” considering the temporal association with drug administration, resolution of symptoms upon discontinuation, and recurrence upon rechallenge. The absence of an alternative explanation further supports this causality. This assessment underscores the importance of systematically evaluating suspected ADRs, particularly in cases requiring drug rechallenge, to ensure accurate identification while prioritizing patient safety.

Management involves discontinuing the offending drug and providing symptomatic treatment, which in this case included corticosteroids and alternative antitubercular medications. Specialist consultations ensured proper care.

After managing the acute reaction, long-term monitoring is crucial to prevent complications such as exfoliative dermatitis, relapse, and the development of drug resistance. This case underscores the importance of pharmacovigilance in identifying and managing ADRs. Reporting to national pharmacovigilance programs like the PvPI plays a vital role in enhancing patient safety and improving therapeutic outcomes. Accordingly, this case was reported to the Adverse Drug Reaction Monitoring Centre under the PvPI at BLDE (Deemed to be University), Shri BM Patil Medical College Hospital and Research Centre, Vijayapura, India. The worldwide unique ID assigned to this case by the Indian Pharmacopoeia Commission is IN-IPC 300636074.

## Conclusions

Drug-induced lichenoid photosensitivity, although rare, remains a clinically significant adverse effect of ATT that can complicate treatment adherence and outcomes. Early recognition, careful differential diagnosis, and systematic drug rechallenge are essential for accurate identification and effective management. Although the diagnosis was strongly supported by clinical findings and drug rechallenge outcomes, the absence of confirmatory tests such as biopsy or photopatch testing represents a limitation of this report. Future cases would benefit from such investigations to further substantiate the diagnosis. This case reinforces the need for heightened clinical vigilance and individualized therapeutic approaches in patients presenting with atypical cutaneous reactions during ATT. Moreover, it underscores the vital role of pharmacovigilance in capturing and analyzing such uncommon reactions, ultimately contributing to safer prescribing practices and improved patient care. Encouraging active ADR reporting and strengthening surveillance mechanisms can play a transformative role in reducing preventable drug-related morbidity.
